# Disentangling shared and unique effects of parenting on psychopathology: Evidence from two prospective, genetically sensitive cohort studies

**DOI:** 10.1002/jcv2.70134

**Published:** 2026-06-05

**Authors:** Leah S. Richmond‐Rakerd, Lara Khalifeh, S. Alexandra Burt, Jody M. Ganiban, Rachel E. Hsu, Luke W. Hyde, Sujin Lee, Leslie D. Leve, Misaki N. Natsuaki, Jenae M. Neiderhiser, David Reiss, Daniel S. Shaw, Gabriela L. Suarez, Jasmin Wertz

**Affiliations:** ^1^ Department of Psychology University of Michigan Ann Arbor Michigan USA; ^2^ Department of Psychology Michigan State University East Lansing Michigan USA; ^3^ Department of Psychological & Brain Sciences The George Washington University Washington District of Columbia USA; ^4^ Department of Psychology University of Texas at Austin Austin Texas USA; ^5^ Survey Research Center at the Institute for Social Research University of Michigan Ann Arbor Michigan USA; ^6^ Department of Psychiatry Michigan Medicine Ann Arbor Michigan USA; ^7^ Prevention Science Institute University of Oregon Eugene Oregon USA; ^8^ Department of Psychology University of California Riverside California USA; ^9^ Department of Psychology Penn State University University Park Pennsylvania USA; ^10^ Child Study Center Yale University New Haven Connecticut USA; ^11^ Department of Psychology University of Pittsburgh Pittsburgh Pennsylvania USA; ^12^ Department of Psychology University of California‐Irvine Irvine California USA; ^13^ Department of Psychology University of Edinburgh Edinburgh UK

**Keywords:** externalizing, genetics, internalizing, longitudinal, parenting, p‐factor, psychopathology, transdiagnostic

## Abstract

**Background:**

Multiple dimensions of parenting–including hostility, conflict, and warmth–are associated with an array of mental disorders in youth. We tested two possible explanations for these associations: Parenting may shape a shared liability to experience many forms of psychopathology (“shared‐liability” hypothesis), or parenting may shape unique liabilities to externalizing and internalizing psychopathology (“unique‐liability” hypothesis).

**Methods:**

Participants were from the Early Growth and Development Study (EGDS; *N* = 561 adoptive families) and the Michigan Twin Neurogenetics Study (MTwiNS; *N* = 354 twin families). In EGDS, we measured parental hostility and warmth at child age 27 months. In MTwiNS, we measured parent‐child conflict and parental involvement at child mean age 8.1 years. We extracted shared and unique liabilities from child‐psychopathology data using structural equation models. We tested whether parenting longitudinally predicted psychopathology, and to what extent associations between parenting and children's externalizing and internalizing psychopathology liabilities were explained by associations with a general psychopathology liability (“p”). We addressed genetic confounding by testing associations of adoptive parents' parenting with psychopathology in their genetically unrelated children, and by using a twin‐difference design.

**Results:**

Across ages and cohorts, parenting characterized by hostility and conflict was associated with greater p, consistent with the shared‐liability hypothesis. Also consistent, parental warmth was associated with lower p in middle and late childhood in EGDS participants, though parental involvement was not associated with p in adolescent MTwiNS participants. There was limited support for the unique‐liability hypothesis; only one association (with externalizing psychopathology) remained after accounting for p. In childhood, but not adolescence, findings remained after accounting for genetic confounding.

**Conclusion:**

Parenting behaviors, particularly hostility and conflict, are associated with children's broad vulnerability to psychopathology, and these associations are evident in childhood even after controlling for genetic influences. These findings support parenting interventions as a means to reduce children's liability to diverse mental‐health problems.

## Key Points

**What's Known**

- Parenting behaviors are linked to a broad array of children's mental‐health problems, but it is unclear to what extent these links reflect shared risk for many different types of psychopathology, or unique risk for externalizing or internalizing problems.

**What's New**

- This study shows that hostile/conflictual parenting and, to a lesser extent, warm parenting are transdiagnostic predictors of children's externalizing and internalizing problems.
- In middle and late childhood, associations of these two domains of parenting with children's psychopathology are evident after accounting for genetic risk.

**What's Relevant**

- These results suggest that parenting interventions, particularly early in life, may help to prevent children from experiencing diverse mental‐health problems as they grow older.

## INTRODUCTION

A wealth of research documents associations between various aspects of parenting behaviors and children's psychopathology (Pinquart, 2017a, 2017b; Yap & Jorm, 2015). For example, parenting characterized by hostility, criticism, and conflict with the child is typically associated with greater risk for children's externalizing (e.g., conduct disorder) and internalizing disorders (e.g., depression), whereas parenting characterized by affection, support, and interest in the child is typically associated with lower risk for children's psychopathology. A key finding from these studies is that parenting is typically associated with both externalizing and internalizing disorders, rather than any one particular type of psychopathology (Aitken et al., 2024; Conway et al., 2018; Ormel et al., 2024).

There are at least two hypotheses that could account for this finding. First, parenting may influence a general liability to develop different types of psychopathology. For example, parenting may predict features or etiological processes that are shared across externalizing and internalizing disorders, such as children's difficulties with emotion regulation (Compas et al., 2017). We refer to this here as the “shared‐liability” hypothesis. Second, parenting may influence more specific liabilities to develop externalizing versus internalizing disorders. For example, parenting may predict features or etiological processes that are uniquely linked to either externalizing or internalizing disorders, such as low effortful control, which tends to be particularly associated with risk for externalizing problems; as well as fear, which tends to be particularly associated with risk for internalizing problems (Ormel et al., 2005). We refer to this here as the “unique‐liability” hypothesis. Testing to what extent parenting predicts a general vulnerability to psychopathology versus unique symptom domains can inform theories about the causes of psychopathology (McLaughlin et al., 2020) and guide research into transdiagnostic versus specific mechanisms linking parenting with psychopathology. It can also inform the design of interventions to reduce children's psychopathology, including those aiming to integrate common elements from different evidence‐based parenting interventions (Marchette & Weisz, 2017). We addressed this question in two genetically‐informative, longitudinal cohorts which together capture developmental periods from early childhood to adolescence.

Our approach to operationalizing shared versus unique liabilities was guided by insights from structural models of psychopathology, which analyze correlations between symptoms to explore higher‐level factors that can statistically account for the observation that many individuals who experience symptoms of one mental disorder also experience symptoms of others (Caspi et al., 2020; Smith et al., 2020). These models provide evidence for a higher‐level factor of psychopathology termed the “general‐psychopathology” or “p”‐factor (Caspi et al., 2014; Lahey et al., 2012), which has been interpreted to reflect a general liability to develop many forms of psychopathology. Despite methodological and conceptual debates concerning the p‐factor (Bornovalova et al., 2020; Waldman et al., 2023; Watts et al., 2024), analyses suggest that it emerges across different modeling strategies (Caspi et al., 2024; Clark et al., 2021; Forbes et al., 2021; Scopel Hoffmann et al., 2022) and replicates across ages, cultures, and informants (Laceulle et al., 2015; Martel et al., 2017), suggesting that it is a relatively robust summary of individuals' propensity to develop a range of common mental disorders. The p‐factor can be contrasted with narrower dimensions representing liabilities to more specific types of psychopathology. A widely‐replicated and well‐validated model contains two dimensions, reflecting liabilities to externalizing and internalizing psychopathology (Achenbach, 1966; Achenbach et al., 2016).

Support for the shared‐liability hypothesis would be provided if different parenting dimensions are associated with the p‐factor, but no longer with externalizing and internalizing psychopathology once the p‐factor is controlled for. Support for the unique‐liability hypothesis would be provided if associations between parenting dimensions and externalizing and internalizing psychopathology remain even after controlling for the p‐factor. Similar tests have been conducted for other predictors of children's psychopathology, including maltreatment exposure, childhood economic disadvantage, and family psychiatric history, often showing evidence for the shared‐liability hypothesis (Caspi et al., 2014, 2024; Conway et al., 2018; McLaughlin et al., 2020). However, few studies have tested these hypotheses in relation to parenting, particularly in early childhood (Aitken et al., 2024; Ormel et al., 2005, 2024). Our study incorporated three features that extend prior research. First, while prior research has largely focused on mothers' parenting (Parent et al., 2017), we additionally included measures of fathers' parenting. Second, we included two cohorts that covered different ages to test whether associations between different dimensions of parenting and children's liabilities for psychopathology are evident across developmental periods. Third, we accounted for genetic confounding of associations between parenting and child outcomes (Hart et al., 2021; Knafo & Jaffee, 2013). Genetic confounding can arise if genes that shape parents' behaviors are transmitted to children and shape children's risk for psychopathology (passive *r*GE) (Wertz et al., 2019). For instance, parents who transmit genetic risk for aggressive behavior to their children may also be more likely to use physical discipline. Genetic confounding can also arise if children at higher genetic risk for psychopathology evoke different parenting (evocative *r*GE) (Klahr & Burt, 2014). For instance, if a child is irritable, a genetically‐influenced characteristic, their parents may respond with negativity. We addressed genetic confounding using a parent‐offspring adoption design and a twin‐difference design. The adoption design controls for passive *r*GE. However, this design does not fully address evocative *r*GE, as adopted children's genetically‐influenced traits can evoke different parenting from their genetically‐unrelated adoptive parents. The twin‐difference design, when restricted to genetically identical (monozygotic; MZ) twins, additionally controls for evocative *r*GE, as MZ twins are matched on their genetic propensity toward psychopathology.

Our study had two aims. First, to test associations of parenting behaviors with children's liabilities to psychopathology in middle childhood, late childhood, and adolescence. We hypothesized that parenting would be associated with the p‐factor, and that associations of parenting with externalizing and internalizing psychopathology would be reduced after accounting for p, consistent with the shared‐liability hypothesis. The second aim was to test whether associations were environmentally mediated, that is, evident after accounting for genetic confounding. We hypothesized that parenting behaviors would be associated with children's psychopathology liabilities even after controlling for genetic confounding.

## METHODS

### Participants

#### Early Growth and Development Study

Participants in the first sample were members of the Early Growth and Development Study (EGDS), a multisite longitudinal study of linked families including 561 adopted children (57.2% male), their adoptive parents (*n* = 569 adoptive mothers, *n* = 562 adoptive fathers, *n* = 41 same‐sex parent families), and their biological mothers (*n* = 554) and fathers (*n* = 210). Numbers do not sum to 561 adoptive mothers/fathers because the sample includes 41 same‐sex parent families, and 11 additional adoptive fathers and 3 additional adoptive mothers who entered the family after the original couple adopted the child. The EGDS sample was recruited in two cohorts (Leve et al., 2013, 2019). The mean age of adopted children at placement was 6 days (SD = 12). EGDS children are relatively racially and ethnically diverse (55.3% White, 19.6% multiracial, 13.2% Black, 10.9% Hispanic or Latinx). EGDS shows the typical pattern of differences in sociodemographic characteristics often found between birth and adoptive parents, with adoptive parents having more advantaged socioeconomic backgrounds than birth parents (DeFries et al., 1994; Natsuaki et al., 2019). Analyses of attrition in EGDS at child ages 7–11 years (the ages for outcome measures in the current study) show no evidence of systematic missingness (Ganiban et al., 2021; Natsuaki et al., 2021), with a few minor exceptions (e.g., higher adoption openness for families with missing data) (Cioffi et al., 2021; Reiss et al., 2022).

#### Michigan Twin Neurogenetics Study

Participants in the second sample included 708 twins in 354 families assessed during early adolescence (hereafter Wave 2) in the Michigan Twin Neurogenetics Study (MTwiNS). MTwiNS families were recruited from the Twin Study of Behavioral and Emotional Development–Child (TBED‐C; hereafter Wave 1), a project within the broader Michigan State University Twin Registry (Burt & Klump, 2019). The TBED‐C included a population‐based arm (528 twin families) with children aged 6–10 years, and an “under‐resourced” arm (502 twin families) from the same geographic region, but residing in neighborhoods with above‐average levels of poverty (neighborhood poverty was ≥10.5%) (Burt & Klump, 2019). As part of a follow‐up neuroimaging study, MTwiNS recruited families from the “under‐resourced” arm and those in the population‐based arm that would have met criteria for the under‐resourced arm (i.e., living in neighborhoods with above‐average levels of poverty). The current MTwiNS sample includes 708 twins in 354 families (mean Wave 2 age = 14.6 years; 54.5% boys; 78.3% White, 13.0% African‐American, 1.1% Latinx, 0.9% Native American, 0.9% Pacific Islander, 0.6% Asian, 5.4% multiracial).

### Measures

#### Self‐reported parenting

In EGDS, parenting was assessed using adoptive parents' self‐reports on the Iowa Hostility and Warmth scales at child age 27 months (Melby & Conger, 2000). Parents used a seven‐point scale (ranging from *never* to *always*) to rate aspects of hostility (5 items, e.g., “Shout or yell at [child] because you were mad at him/her”) and warmth (4 items, e.g., “Act loving and affectionate toward [child]”). The scales show good internal consistency in EGDS at the 27‐month assessment (Elam et al., 2014; Reuben et al., 2016). Adoptive mothers' and fathers' reports of their parenting were correlated (hostility: *r* = 0.33, *p* < .0001; warmth: *r* = 0.23, *p* < .0001), and so their ratings were averaged (for sensitivity analyses using each parent's ratings separately, see Table S1). When data were available from only one parent, the rating was taken from that parent (more often the mother). Of EGDS participants with parenting data, 92% had data from both parents. The averaged hostility and warmth ratings were inversely correlated (*r* = −0.33, *p* < .0001).

In MTwiNS, parenting was assessed using parents' self‐reports on the Parent Environment Questionnaire (PEQ) Conflict and Involvement scales at Wave 1 (child mean age 8.1 years). The PEQ assesses factorially‐derived aspects of the relationship of each parent‐child dyad in the family (Elkins et al., 1997). Parents used a four‐point scale (ranging from *definitely false* to *definitely true*) to rate aspects of conflict (12 items, e.g., “I often criticize my child”) and involvement (12 items, e.g., “I praise my child when he/she does something well”) in the parent‐child relationship. The PEQ Conflict and Involvement scales show good internal consistency in MTwiNS (Dotterer et al., 2021). Mothers' and fathers' reports were correlated (conflict: *r* = 0.28, *p* < .0001; involvement: *r* = 0.20, *p* < .001), and so their ratings were averaged (for sensitivity analyses using each parent's ratings separately, see Table S2). When data were available from only one parent, the rating was taken from that parent (more often the mother). Of MTwiNS participants with parenting data, 79% had data from both parents. The averaged conflict and involvement ratings were inversely correlated (*r* = –0.25, *p* < .0001).

#### Child psychopathology

In EGDS and MTwiNS, children's externalizing and internalizing problems were assessed using the Child Behavior Checklist (CBCL) (Achenbach, 1978). In EGDS, child psychopathology was measured at ages 27 months, 7 years, and 11 years using adoptive‐mother and adoptive‐father reports. At 27 months, parents completed the preschool version of the CBCL (CBCL/1.5–5) (Achenbach & Rescorla, 2000); at 7 and 11 years, parents completed the school‐age version (CBCL/6–18) (Achenbach & Rescorla, 2001). In MTwiNS, mothers and fathers completed the school‐age version at Wave 1 (child mean age 8.1 years) and Wave 2 (child mean age 14.6 years).

CBCL items were rated on a three‐point scale: “not true,” “somewhat/sometimes true,” and “very true/often.” CBCL items were prepared for analyses as follows: First, due to low endorsement of the “very true/often” category, we recoded items into a binary format by combining the “somewhat/sometimes true” and “very true/often” categories. This is consistent with prior item‐level analyses of the CBCL, including analyses of the CBCL factor structure, across a range of samples (Dang et al., 2017; Deutz et al., 2016; Dumenci et al., 2004; Ivanova et al., 2007; McElroy et al., 2018). Second, we combined ratings by mothers and fathers, coding an item as endorsed if either parent endorsed it. When data were available from only one parent, the rating was taken from that parent. Third, to support model estimation, we removed items with very low endorsement (prevalences below 5%) and items with empty cell counts when combined with other items (Table S3A,B).

#### Covariates

In EGDS, covariates included child sex, child race (0 = White, 1 = Non‐White), adoption openness, and obstetric complications. Adoption openness was measured as the degree of contact, disclosure, and frequency of communication between birth parents and adoptive families, at ages 4–9 months. We used a standardized mean of birth‐mother, adoptive‐mother, and adoptive‐father ratings regarding the extent to which they perceived the adoption was open on a seven‐point scale (Ge et al., 2008). We used a composite measure of obstetric complications comprising a comprehensive assessment of perinatal complications from birth mothers from data obtained by a pregnancy screener and a pregnancy calendar method developed for the study (originating from (Caspi et al., 1996)). It included data on maternal risk factors (e.g., depressive mood) and pregnancy complications (e.g., drug exposure), labor and delivery complications (e.g., prolonged labor), and neonatal complications (e.g., prematurity) (Marceau et al., 2013).

In MTwiNS, covariates included child sex, child race (0 = White, 1 = Non‐White), and baseline age.

### Statistical analyses

#### Factor modeling

We used confirmatory factor analysis (CFA) of items to test two standard models representing the structure of psychopathology: a correlated‐factors model and a bifactor model (Shields et al., 2019; Waldman et al., 2016). In the correlated‐factors model, CBCL items loaded onto either externalizing or internalizing factors, and the factors were allowed to correlate. The factor loadings in this model indicate to what extent each item reflects externalizing or internalizing psychopathology. In the bifactor model, items loaded onto a general‐psychopathology factor (“p”) and onto either externalizing or internalizing sub‐factors. The sub‐factors were uncorrelated with each other and uncorrelated with the p‐factor (Watts et al., 2024). Factor loadings on the p‐factor indicate to what extent each item reflects general psychopathology. Factor loadings on the specific factors indicate to what extent each item reflects a more narrow style of either externalizing or internalizing psychopathology, net of general psychopathology.

We used a bifactor model rather than a higher‐order model (in which items load onto externalizing and internalizing factors, which load onto a p‐factor) because the bifactor model is better suited to testing general and specific associations between factor scores and an external variable (Lahey et al., 2021), the goal of this study. Additionally, a higher‐order model with only two sub‐factors is not identified without additional constraints. However, sensitivity analyses indicated that factor scores for the p‐factor from the bifactor model and from a one‐factor model were very highly correlated. In EGDS, the scores were correlated *r* = 0.96, 0.98, and 0.94 at ages 27 months, 7 years, and 11 years, respectively. In MTwiNS, the scores were correlated *r* = 0.94 and 0.96 at ages 8.1 and 14.6 years, respectively. This aligns with prior work indicating that p performs similarly regardless of how it is modeled (Caspi et al., 2024).

CFA analyses were performed in MPlus (Muthén & Muthén, 1998–2017) using the weighted least squares means and variance adjusted (WLSMV) algorithm. CFA analyses in MTwiNS accounted for the clustering of twins within families. We exported the factor scores from the correlated‐factors and bifactor models and used these in tests of association with parenting measures.

#### Tests of association

In EGDS, we used linear regression to test longitudinal associations of adoptive‐parent parenting in early childhood (age 27 months) with child psychopathology in middle childhood (7 years) and late childhood (11 years), before and after extracting p. Models controlled for baseline psychopathology (e.g., when predicting the externalizing factor at age 7 years, we controlled for the externalizing factor at age 27 months), child sex, child race, adoption openness, and obstetric complications.

In MTwiNS, we used linear regression (with standard errors adjusted for the clustering of twins within families) to test longitudinal associations between parenting in middle childhood (child mean age 8.1 years) and child psychopathology in adolescence (mean age 14.6 years). We tested associations before and after extracting p. Models controlled for baseline child psychopathology (as in EGDS), child sex, child race, and baseline child age. For models where tests indicated significant associations of parenting with child psychopathology, we additionally used fixed‐effects models to conduct a twin‐difference analysis, to test whether twins who were parented differently also differed in their levels of psychopathology, net of their shared family background. If so, and if this persists within genetically identical twin pairs, this would suggest that parenting effects are environmentally mediated, that is, independent of genetic influences. Fixed‐effects models controlled for baseline child‐psychopathology levels and child sex (because opposite‐sex twins were included). We did not control for race or baseline age as twins were matched on these characteristics within pairs.

We analyzed participants with complete data across waves. In EGDS, we required that participants have complete data on the two waves considered in each model; participants were not required to have data at all three waves. In EGDS, 386 (of 561; 69%) participants had complete data at ages 27 months and 7 years, and 380 (68%) participants had complete data at 27 months and 11 years. In MTwiNS, 699 (of 708; 99%) participants had complete data at ages 8.1 and 14.6 years. Analyses were conducted using SAS version 9.4.

## RESULTS

### Modeling the structure of child psychopathology

In EGDS, the correlated‐factors model and the bifactor model fit the data well (Table 1). In the correlated‐factors model, item loadings on the externalizing and internalizing factors were all positive, averaging 0.63–0.70 for externalizing problems and 0.51–0.60 for internalizing problems, depending on age. Correlations between the two factors were 0.75, 0.67, and 0.76 at ages 27 months, 7 years, and 11 years, respectively. In the bifactor model, item loadings on the general‐psychopathology factor (p) were all positive, averaging 0.48 (27 months)–0.56 (11 years). Loadings for the specific factors were reduced compared to the correlated‐factors model, averaging 0.22 (7 years)–0.31 (11 years), consistent with the hypothesis that the p‐factor accounts for a portion of the variance in these items.

**TABLE 1:** Fit statistics of the correlated‐factors and bifactor (p‐factor) models at each child age in the EGDS and MTwiNS samples.

Age	Model	*Χ* ^2^	*df*	CFI	TLI	RMSEA
EGDS sample^a^						
27 months	Correlated	1698.046	1033	0.90	0.90	0.04
	p‐factor	1421.792	988	0.94	0.93	0.03
7 years	Correlated	2492.249	1594	0.89	0.89	0.04
	p‐factor	2116.924	1538	0.93	0.93	0.03
11 years	Correlated	2960.101	1768	0.91	0.91	0.04
	p‐factor	2475.034	1708	0.94	0.94	0.03
MTwiNS sample^b^						
Mean = 8.1 years	Correlated	3801.625	2014	0.83	0.83	0.04
	p‐factor	3050.020	1950	0.90	0.89	0.03
Mean = 14.6 years	Correlated	3478.188	1828	0.88	0.87	0.04
	p‐factor	2816.937	1767	0.92	0.92	0.03

^a^ *n* = 500, 420, and 409 EGDS participants with CBCL data at ages 27 months, 7 years, and 11 years, respectively.

^b^ *n* = 706 and 705 MTwiNS participants with CBCL data at mean age 8.1 years and mean age 14.6 years, respectively.

In MTwiNS, the correlated‐factors model and the bifactor model generally fit the data well, but model‐fit indices, particularly for the correlated‐factors model at age 8.1 years, were slightly poorer than for EGDS (Table 1). In the correlated‐factors model, item loadings on the externalizing and internalizing factors were all positive, averaging 0.63 at age 8.1 years and 0.68 at age 14.6 years for externalizing problems, and 0.53 at age 8.1 years and 0.61 at age 14.6 years for internalizing problems. Correlations between the factors were 0.60 and 0.65 at ages 8.1 and 14.6 years, respectively. In the bifactor model, item loadings on the p‐factor were all positive, averaging 0.46 and 0.53 at ages 8.1 and 14.6 years, respectively. Loadings for the specific factors were reduced compared to the correlated‐factors model, averaging 0.32 and 0.15 at ages 8.1 and 14.6 years, respectively.

### Stability of psychopathology dimensions across age

In EGDS, cross‐age correlations for the derived psychopathology dimensions ranged from 0.37–0.70 for externalizing problems (from the correlated‐factors model), 0.33–0.62 for internalizing problems (from the correlated‐factors model), and 0.34–0.57 for the p‐factor. In MTwiNS, cross‐age correlations were 0.51 for externalizing problems (from the correlated‐factors model), 0.44 for internalizing problems (from the correlated‐factors model), and 0.53 for the p‐factor.

### Longitudinal associations of parenting in early childhood with children's psychopathology in middle childhood, in the EGDS sample

In EGDS, we tested whether adoptive parents' reports of their parenting at age 27 months were associated with children's externalizing and internalizing psychopathology at age 7 years, using psychopathology dimensions from the correlated‐factors model. Unadjusted associations are shown in Table S4. After controlling for children's baseline psychopathology at 27 months, child sex, child race, adoption openness, and obstetric complications, more hostile parenting at 27 months predicted more externalizing (*β* = 0.18 [0.08, 0.27]) and internalizing (*β* = 0.16 [0.07, 0.26]) problems at 7 years (Table 2), whereas warmer parenting at 27 months predicted fewer externalizing (*β* = −0.13 [−0.23, −0.04]) and internalizing (*β* = −0.11 [‐0.21, −0.01]) problems at 7 years (Table 2). After extracting p, these associations were reduced and became non‐significant (Table 2). In these models, adoptive‐parent hostility and warmth were associated with child p (hostility: *β* = 0.23 [0.14, 0.33]; warmth: *β* = −0.19 [−0.28, −0.09]; Table 2), but no longer with externalizing and internalizing psychopathology. These findings are consistent with the shared‐liability hypothesis: Hostile and warm parenting in early childhood predict children's risk for psychopathology in middle childhood via a general liability to psychopathology. Associations were evident for adoptive parents' parenting, ruling out passive *r*GE.

**TABLE 2:** Longitudinal associations of self‐reported parenting with child psychopathology dimensions from correlated‐factors and bifactor (p‐factor) models, across developmental periods.

	Developmental period of psychopathology outcome measures					
	Middle childhood (EGDS sample)^a^		Late childhood (EGDS sample)^a^		Adolescence (MTwiNS sample)^a^	
	Correlated	p‐factor	Correlated	p‐factor	Correlated	p‐factor
Hostility (EGDS) and conflict (MTwiNS)						
Externalizing	**0.18 [0.08, 0.27]**	−0.03 [−0.14, 0.07]	**0.20 [0.10, 0.30]**	**0.16 [0.06, 0.26]**	0.09 [−0.003, 0.19]	0.02 [−0.05, 0.10]
Internalizing	**0.16 [0.07, 0.26]**	0.01 [−0.08, 0.11]	**0.16 [0.06, 0.27]**	−0.06 [−0.16, 0.05]	**0.11 [0.03, 0.20]**	0.06 [−0.02, 0.13]
p	‐‐	**0.23 [0.14, 0.33]**	‐‐	**0.18 [0.07, 0.28]**	‐‐	**0.10 [0.01, 0.18]**
Warmth (EGDS) and involvement (MTwiNS)						
Externalizing	**−0.13 [**−**0.23, −0.04]**	0.10 [−0.004, 0.20]	**−0.11 [−0.21, −0.01]**	−0.07 [−0.17, 0.03]	−0.06 [−0.13, 0.02]	−0.01 [−0.08, 0.07]
Internalizing	**−0.11 [**−**0.21, −0.01]**	0.01 [−0.08, 0.11]	−0.10 [−0.20, 0.004]	0.01 [−0.09, 0.11]	−0.07 [−0.15, 0.004]	−0.05 [−0.12, 0.02]
p	‐‐	**−0.19 [**−**0.28,** −**0.09]**	‐‐	**−0.11 [**−**0.21, −0.01]**	‐‐	−0.05 [−0.13, 0.02]

*Note*: Estimates are standardized regression coefficients. Models in the EGDS sample controlled for baseline psychopathology (the parallel psychopathology dimension at age 27 months), child sex, child race, adoption openness, and obstetric complications. Models in the MTwiNS sample controlled for baseline psychopathology (the parallel psychopathology dimension at age 8.1 years), child sex, child race, and baseline child age. In the MTwiNS sample, standard errors were adjusted for the clustering of twins within families. Brackets indicate 95% confidence intervals. Bolded estimates are statistically significant.

^a^ Middle‐childhood models predicted child psychopathology at age 7 years from parenting at age 27 months (*n* = 386 with complete data). Late‐childhood models predicted child psychopathology at age 11 years from parenting at age 27 months (*n* = 380 with complete data). Adolescence models predicted child psychopathology at mean age 14.6 years from parenting at mean age 8.1 years (*n* = 699 with complete data).

### Longitudinal associations of parenting in early childhood with children's psychopathology in late childhood, in the EGDS sample

In EGDS, we next tested whether adoptive parents' reports of their parenting at age 27 months were associated with children's externalizing and internalizing psychopathology at age 11 years, using psychopathology dimensions from the correlated‐factors model. Unadjusted associations are shown in Table S4. After controlling for baseline psychopathology at age 27 months, child sex, child race, adoption openness, and obstetric complications, more hostile parenting at 27 months predicted more child externalizing (*β* = 0.20 [0.10, 0.30]) and internalizing (*β* = 0.16 [0.06, 0.27]) problems at 11 years (Table 2), whereas warmer parenting at 27 months predicted fewer externalizing problems at 11 years (*β* = −0.11 [−0.21, −0.01]). Parental warmth did not significantly predict children's internalizing problems (Table 2).

Consistent with findings from early childhood, after extracting p, associations between parental hostility and children's psychopathology were reduced. Parental hostility no longer significantly predicted children's internalizing problems, but the association with externalizing problems remained significant (*β* = 0.16 [0.06, 0.26]); and parental warmth no longer significantly predicted children's externalizing problems (Table 2). Also consistent with findings from early childhood, parental hostility and warmth were associated with child p (hostility: *β* = 0.18 [0.07, 0.28]; warmth: *β* = −0.11 [−0.21, −0.01]; Table 2). These results align with both the shared‐ and unique‐liability hypotheses: Hostile and warm parenting in early childhood predict children's risk for psychopathology in late childhood via a general liability to psychopathology, as well as associations with a unique liability to externalizing psychopathology. Associations were evident for adoptive parents' parenting, ruling out passive *r*GE.

### Longitudinal associations of parenting in middle childhood with children's psychopathology in adolescence, in the MTwiNS sample

In MTwiNS, we tested whether parents' reports of conflictual parent‐child relationships and involved parenting at average age 8.1 years were associated with children's externalizing and internalizing psychopathology at average age 14.6 years, using psychopathology dimensions from the correlated‐factors model. Unadjusted associations are shown in Table S5. After controlling for baseline psychopathology at age 8.1 years, child sex, child race, and baseline child age, more parent‐child conflict at age 8.1 years predicted more internalizing problems at age 14.6 years (*β* = 0.11 [0.03, 0.20]), but the association with externalizing problems was not statistically significant (Table 2). Parental involvement at 8.1 years did not significantly predict child externalizing or internalizing problems at 14.6 years (Table 2).

Consistent with findings from middle and late childhood in EGDS, after extracting p, parent‐child conflict no longer significantly predicted children's internalizing psychopathology (Table 2). Also consistent, parent‐child conflict significantly predicted child p (*β* = 0.10 [0.01, 0.18]; Table 2). Parental involvement was not significantly associated with any psychopathology dimensions (Table 2). These results are consistent with the shared‐liability hypothesis: Parenting in middle childhood predicts children's risk for psychopathology in adolescence via a general psychopathology liability.

Where we observed significant associations between parenting and children's psychopathology in MTwiNS, we used fixed‐effects models to test whether these associations held within twin pairs. Dizygotic (DZ) twins who differed in their levels of parent‐child conflict also differed in their levels of internalizing psychopathology (from the correlated‐factors model; *β* = 0.25 [0.08, 0.43]) and the p‐factor (*β* = 0.20 [0.02, 0.38]). However, these associations did not hold within MZ pairs (internalizing psychopathology: *β* = −0.02 [−0.24, 0.21]; p‐factor: *β* = −0.05 [−0.33, 0.23]), indicating that associations of parenting in middle childhood with children's psychopathology in adolescence are largely explained by genetic confounding.

## DISCUSSION

Our analysis of two prospective, genetically sensitive cohorts yielded three key findings regarding associations between different dimensions of parenting and children's liabilities to psychopathology. First, across developmental periods and type of design (adoption and twin), there was consistent support for the shared‐liability hypothesis, suggesting that parenting is associated with children's psychopathology in part via a general liability to psychopathology. In contrast, evidence for the unique‐liability hypothesis was less consistent and restricted to one association, between hostile parenting and externalizing psychopathology. Second, parenting characterized by hostility and conflict showed more consistent associations with children's psychopathology compared to parenting characterized by warmth and involvement. Third, the evidence concerning environmental mediation of parenting effects was conflicting, potentially due to variation in samples' study designs or developmental phases: Findings from the adoption design in middle and late childhood provided support for an environmentally‐mediated effect of parenting, but this effect was not evident in adolescence using the twin design.

Our findings should be interpreted in light of limitations. First, parents reported on both their parenting and their children's psychopathology, thus, shared informant and method bias might have inflated associations. However, we combined mothers' and fathers' reports to reduce the impact of individual rater biases. Second, the cohorts differed in several ways, including parenting measures and parenting factors used, participant ages, ages when parenting was measured, and the types of environmental influences measured in their designs (both shared and unique environmental influences in the adoption design, but only unique environmental influences in the twin‐difference design). Additionally, EGDS adoptive families are economically advantaged, while MTwiNS families have a higher proportion living in under‐resourced neighborhoods. This enabled us to capture a broader range of socioeconomic diversity, but may have contributed to variation in findings across samples and limits generalizability to samples with different characteristics. Third, the adoption design does not fully account for evocative *r*GE. However, we used cross‐lagged analyses to control for children's earlier psychopathology, which may evoke differences in parental treatment. Fourth, although there was variability in levels of externalizing and internalizing problems in these community samples, results may differ in clinical samples with higher levels of psychopathology. Lastly, our use of longitudinal data, controls for baseline psychopathology levels, and quasi‐experimental, genetically‐informative methods all enhance causal inference, but our analyses are observational and cannot confirm parenting as a causal factor for children's psychopathology.

Notwithstanding limitations, our study has several implications. Our findings showing associations between parenting and children's shared liability for psychopathology support the search for transdiagnostic risk factors that parenting may shape, such as problems in self‐regulation and socio‐emotional functioning (Aldao et al., 2016; Dadds & Frick, 2019; Lynch et al., 2021; Nolen‐Hoeksema et al., 2011). Although we are unable to draw strong inferences about developmental differences due to the confounding of developmental period with cohort, the evidence for shared liability was most obvious for parenting in early childhood predicting psychopathology in middle childhood, when associations between parenting and psychopathology were accounted for by p. As children grew older, parental hostility was additionally associated with children's unique risk for externalizing problems in the EGDS sample, which is consistent with previous work identifying unique associations between parenting and externalizing problems, over and above p (Aitken et al., 2024; Brislin et al., 2022). For future research, this raises the question of how parenting may influence unique risk for externalizing problems, such as via affecting children's emotional attention, responsiveness to others' distress, and responsiveness to potential punishment (Frick et al., 2014; Lynch et al., 2021; Waller et al., 2012, 2013).

For interventions, our findings suggest that efforts to support parenting practices may have broad effects on children's risk for a variety of problem behaviors. In addition to parenting interventions, these efforts might also include treatments to address upstream or co‐occurring problems that shape parenting behavior; for instance, parents' own mental health (Lawrence et al., 2025). Efforts may be most effective when implemented in early childhood, as suggested by our finding that associations between parenting at this age and psychopathology in middle childhood were (a) observed for hostility and conflict as well as warmth and involvement, (b) evident for both externalizing and internalizing problems, and (c) accounted for by associations between parenting and children's liability to general psychopathology, consistent with transdiagnostic effects (Tein et al., 2023). Further, associations remained after controlling for a key confounder—genetic transmission from parent to child—strengthening causal inference. Together with evidence that parenting behaviors and children's externalizing and internalizing problems are malleable (De Graaf et al., 2008; Jeong et al., 2021; Jugovac et al., 2022; Tully & Hunt, 2016; Zarakoviti et al., 2021), these findings lend further support to the view that early childhood may be a critical and fruitful intervention window (Heckman, 2006) for the development and delivery of interventions to reduce children's mental‐health problems.

## AUTHOR CONTRIBUTIONS

**Leah S. Richmond‐Rakerd**: Writing—original draft; visualization; writing—review and editing; formal analysis; conceptualization; supervision. **Lara Khalifeh**: Formal analysis; writing—review and editing. **S. Alexandra Burt**: Writing—review and editing; funding acquisition; methodology; data curation; project administration; resources. **Jody M. Ganiban**: Funding acquisition; methodology; validation; project administration; data curation; resources; writing—review and editing; supervision. **Rachel E. Hsu**: Writing—review and editing; formal analysis. **Luke W. Hyde**: Data curation; resources; project administration; methodology; writing—review and editing; funding acquisition. **Sujin Lee**: Formal analysis; writing—review and editing. **Leslie D. Leve**: Funding acquisition; methodology; data curation; resources; project administration; writing—review and editing. **Misaki N. Natsuaki**: Resources; data curation; writing—review and editing; funding acquisition; project administration; methodology. **Jenae M. Neiderhiser**: Methodology; funding acquisition; data curation; resources; project administration; writing—review and editing. **David Reiss**: Writing—review and editing; supervision; resources; data curation; project administration; funding acquisition; conceptualization; methodology. **Daniel S. Shaw**: Methodology; funding acquisition; data curation; resources; project administration; writing—review and editing. **Gabriela L. Suarez**: Writing—review and editing; formal analysis. **Jasmin Wertz**: Supervision; writing—original draft; conceptualization; writing—review and editing; visualization; formal analysis.

## CONFLICT OF INTEREST STATEMENT

The authors declare no conflicts of interest.

## ETHICAL CONSIDERATIONS

Ethical approval for the MTwiNS study was obtained from the University of Michigan Institutional Review Board (IRB# HUM00163965). Informed consent was obtained from parents and assent was obtained from children. Ethical approval for the EGDS study was obtained from institutional review boards at the University of Oregon (IRB# 0304201400), The Pennsylvania State University (IRB# CR00007591), and George Washington University (IRB# 041119). Informed consent was obtained from adult participants and assent was obtained from children beginning at age 7 years.

## Supporting information

Tables S1–S5

## Data Availability

Anonymized data for the MTwiNS study are shared via the National Institute of Mental Health (NIMH) Data Archive (https://nda‐nih‐gov.proxy.lib.umich.edu/edit_collection.html?id=2818). EGDS data are generally publicly available from NICHD's DASH repository, within the Environmental influences on Child Health Outcomes (ECHO)‐wide Cohort—3rd Release (ECHO Cohorts v3): https://dash.nichd.nih.gov/study/426432. Additional EGDS data can be obtained by contacting the project's data manager (sguyer@uoregon.edu), providing a data analysis proposal, and agreeing to data security terms.
