# Supplementary material for: Disentangling shared and unique effects of parenting on psychopathology: Evidence from two prospective, genetically sensitive cohort studies
Source: JCPP Adv. 2026 Jun 5:e70134. Online ahead of print. doi: 10.1002/jcv2.70134 (PMC13338980; doi:10.1002/jcv2.70134)
Supplement: Supplementary file 1 — Tables S1–S5 [file JCV2-9999-e70134-s001.docx]

**Disentangling shared and unique effects of parenting on psychopathology:**

**Evidence from two prospective, genetically sensitive cohort studies**

**Supporting Information**

**Table S1.** Longitudinal associations of parenting with child psychopathology in the EGDS sample, using maternal and paternal reports of parenting separately.

**Table S2.** Longitudinal associations of parenting with child psychopathology in the MTwiNS sample, using maternal and paternal reports of parenting separately.

**Table S3.** Summary of CBCL items removed due to low endorsement or empty cell counts in the EGDS and MTwiNS samples.

**Table S4.** Raw correlations of parenting at child age 27 months with child psychopathology at ages 27 months, 7 years, and 11 years in the EGDS sample.

**Table S5.** Raw correlations of parenting at child mean age 8.1 years with child psychopathology at mean ages 8.1 and 14.6 years in the MTwiNS sample.

**Table S1.** Longitudinal associations of parenting with child psychopathology in the EGDS sample, using maternal and paternal reports of parenting separately.

|  | Middle childhood | | | | Late childhood | | | |
| --- | --- | --- | --- | --- | --- | --- | --- | --- |
|  | Maternal reports^a^ | | Paternal reports^b^ | | Maternal reports^c^ | | Paternal reports^d^ | |
|  | Correlated | p-factor | Correlated | p-factor | Correlated | p-factor | Correlated | p-factor |
| Hostility |  |  |  |  |  |  |  |  |
| Externalizing | **.14 [.05, .24]** | -.02 [-.13, .08] | **.12 [.03, .22]** | -.02 [-.13, .09] | **.19 [.09, .28]** | **.14 [.04, .24]** | **.13 [.03, .24]** | **.15 [.05, .26]** |
| Internalizing | .09 [-.002, .19] | -.04 [-.13, .06] | **.14 [.04, .24]** | .03 [-.07, .13] | **.15 [.05, .25]** | -.03 [-.13, .07] | .09 [-.02, .19] | -.08 [-.18, .03] |
| p | -- | **.19 [.10, .29]** | -- | **.17 [.07, .27]** | -- | **.17 [.07, .27]** | -- | .10 [-.01, .20] |
| Warmth |  |  |  |  |  |  |  |  |
| Externalizing | **-.12 [-.21, -.02]** | .06 [-.04, .16] | -.07 [-.16, .03] | .10 [-.004, .21] | **-.14 [-.23, -.04]** | -.07 [-.16, .03] | -.01 [-.11, .09] | -.03 [-.13, .07] |
| Internalizing | **-.10 [-.19, -.004]** | .02 [-.07, .12] | -.06 [.16, .04] | .01 [-.09, .11] | **-.14 [-.24, -.04]** | .03 [-.07, .14] | .01 [-.10, .11] | -.02 [-.12, .09] |
| p | -- | **-.16 [-.25, -.07]** | -- | **-.11 [-.21, -.01]** | -- | **-.16 [-.25, -.06]** | -- | -.001 [-.10, .10] |

*Notes.* Estimates are standardized regression coefficients. Middle-childhood models predicted child psychopathology at age 7 years from parenting at age 27 months. Late-childhood models predicted child psychopathology at age 11 years from parenting at age 27 months. Models controlled for baseline psychopathology (the parallel psychopathology dimension at age 27 months), child sex, child race, adoption openness, and obstetric complications. Brackets indicate 95% confidence intervals. Bolded estimates are statistically significant. ^a^n=383 with complete data. ^b^n=361 with complete data. ^c^n=377 with complete data. ^d^n=354 with complete data.

**Table S2.** Longitudinal associations of parenting with child psychopathology in the MTwiNS sample, using maternal and paternal reports of parenting separately.

|  | Maternal reports^a^ | | Paternal reports^b^ | |
| --- | --- | --- | --- | --- |
| Conflict | Correlated | p-factor | Correlated | p-factor |
| Externalizing | .09 [-.01, .18] | .01 [-.06, .09] | .08 [-.01, .16] | .01 [-.08, .09] |
| Internalizing | **.10 [.01, .18]** | .05 [-.03, .13] | **.12 [.03, .21]** | .06 [-.02, .15] |
| p | -- | **.10 [.01, .18]** | -- | .07 [-.01, .16] |
| Involvement |  |  |  |  |
| Externalizing | -.07 [-.15, .01] | .01 [-.07, .09] | -.03 [-.11, .04] | -.02 [-.10, .06] |
| Internalizing | -.05 [-.12, .03] | .01 [-.07, .09] | -.07 [-.16, .01] | -.08 [-.17, .001] |
| p | -- | -.07 [-.15, .01] | -- | -.03 [-.11, .05] |

*Notes.* Estimates are standardized regression coefficients. Models predicted child psychopathology at mean age 14.6 years from parenting at mean age 8.1 years. Models controlled for baseline psychopathology (the parallel psychopathology dimension at age 8.1 years), child sex, child race, and baseline child age. Standard errors were adjusted for the clustering of twins within families. Brackets indicate 95% confidence intervals. Bolded estimates are statistically significant. ^a^n=689 with complete data (conflict models) and n=688 with complete data (involvement models). ^b^n=560 with complete data. **Table S3.** Summary of CBCL items removed due to low endorsement (prevalence < 5%) or empty cell counts in the EGDS and MTwiNS samples.

A. EGDS sample, item number (and endorsement, %)

| Age 27 months | | Age 7 years | | Age 11 years | |
| --- | --- | --- | --- | --- | --- |
| EXT | INT | EXT | INT | EXT | INT |
| 8 (92.4) | 90 (4.8) | 2 (0) | 52 (4.8) | 2 (0) | 56d (4.9) |
| 53 (10.2) | 71 (4.0) | 67 (1.2) | 91 (2.8) | 67 (1.7) | 91 (9.1)^a^ |
|  | 46 (3.5) | 72 (0.7) | 51 (1.9) | 72 (1.7) |  |
|  | 83 (4.0) | 73 (0.2) | 54 (4.8) | 73 (0.5) |  |
|  | 39 (1.4) | 82 (4.2) | 56d (3.3) | 82 (4.7) |  |
|  | 45 (1.2) | 96 (0.2) | 102 (5.4)^a^ | 96 (1.7) |  |
|  | 78 (2.7) | 99 (0) |  | 99 (0) |  |
|  | 93 (3.7) | 101 (0.2) |  | 101 (0.2) |  |
|  | 51 (5.1) | 105 (0) |  | 105 (0) |  |
|  | 47 (6.4)^a^ | 106 (0.1) |  | 106 (2.4) |  |
|  | 67 (6.2)^a^ | 89 (4.1) |  | 3 (81.1)^a^ |  |
|  |  | 37 (5.2)^a^ |  | 37 (6.4)^a^ |  |
|  |  | 22 (78.3)^a^ |  | 22 (68.1)^a^ |  |
|  |  |  |  | 4 (87.8)^a^ |  |

B. MTwiNS sample, item number (and endorsement, %)

| Mean age 8.1 years | | Mean age 14.6 years | |
| --- | --- | --- | --- |
| EXT | INT | EXT | INT |
| 2 (0.1) | 51 (3.1) | 2 (4.3) | 91 (2.0) |
| 67 (1.6) | 91 (6.2)^a^ | 67 (1.3) | 56d (3.8) |
| 72 (0.8) |  | 72 (1.4) | 56g (4.5) |
| 73 (1.6) |  | 73 (0.7) |  |
| 82 (4.4) |  | 82 (2.0) |  |
| 96 (0.7) |  | 96 (1.7) |  |
| 99 (0.1) |  | 99 (1.4) |  |
| 101 (0.1) |  | 101 (2.1) |  |
| 105 (0.0) |  | 105 (2.1) |  |
| 106 (1.3) |  | 106 (0.4) |  |
|  |  | 37 (2.8) |  |
|  |  | 97 (3.5) |  |

^a^Item was excluded due to empty cell counts when combined with other items.

**Table S4.** Raw correlations of parenting at child age 27 months with child psychopathology at ages 27 months, 7 years, and 11 years, in the EGDS sample.

|  | EXT, CF, 27m | INT, CF, 27m | p, BF,  27m | EXT, CF,  7y | INT, CF,  7y | p, BF,  7y | EXT, CF,  11y | INT, CF,  11y | p, BF,  11y |
| --- | --- | --- | --- | --- | --- | --- | --- | --- | --- |
| Hostility, 27m | .41 | .33 | .37 | .33 | .26 | .34 | .31 | .24 | .26 |
| Warmth, 27m | -.31 | -.27 | -.28 | -.27 | -.20 | -.28 | -.22 | -.17 | -.19 |

Note. All estimates are statistically significant. EXT = externalizing, INT = internalizing, CF = correlated-factors model, BF = bifactor model.

**Table S5.** Raw correlations of parenting at child mean age 8.1 years with child psychopathology at mean ages 8.1 and 14.6 years, in the MTwiNS sample.

|  | EXT, CF, 8.1y | INT, CF, 8.1y | p, BF, 8.1y | EXT, CF, 14.6y | INT, CF, 14.6y | p, BF, 14.6y |
| --- | --- | --- | --- | --- | --- | --- |
| Conflict, 8.1y | .51 | .41 | .48 | .33 | .28 | .33 |
| Involvement, 8.1y | -.14 | -.15 | -.15 | -.13 | -.13 | -.13 |

Note. All estimates are statistically significant. EXT = externalizing, INT = internalizing, CF = correlated-factors model, BF = bifactor model.
